# Understanding the Role of Non-Coding RNAs in Bladder Cancer: From Dark Matter to Valuable Therapeutic Targets

**DOI:** 10.3390/ijms18071514

**Published:** 2017-07-13

**Authors:** Cecilia Pop-Bica, Diana Gulei, Roxana Cojocneanu-Petric, Cornelia Braicu, Bogdan Petrut, Ioana Berindan-Neagoe

**Affiliations:** 1 Research Center for Functional Genomics, Biomedicine and Translational Medicine, University of Medicine and Pharmacy “Iuliu-Hatieganu”, Cluj-Napoca 400337, Romania; cecilia.bica8@gmail.com (C.P.-B.); cojocneanur@gmail.com (R.C.-P.); cornelia.braicu@umfcluj.ro (C.B.); 2 MedFuture Research Center for Advanced Medicine, University of Medicine and Pharmacy “Iuliu-Hatieganu”, Cluj-Napoca 400349, Romania; gulei.diana@umfcluj.ro; 3 Department of Urology, The Oncology Institute “Prof Dr. Ion Chiricuta”, Cluj-Napoca 400015, Romania; 4 Department of Urology, “Iuliu Hatieganu” University of Medicine and Pharmacy, Cluj-Napoca 400012, Romania; 5 Department of Functional Genomics and Experimental Pathology, The Oncology Institute “Prof. Dr. Ion Chiricuţă”, Cluj-Napoca 400015, Romania

**Keywords:** bladder cancer, non-coding RNA, target gene, Warburg effect

## Abstract

The mortality and morbidity that characterize bladder cancer compel this malignancy into the category of hot topics in terms of biomolecular research. Therefore, a better knowledge of the specific molecular mechanisms that underlie the development and progression of bladder cancer is demanded. Tumor heterogeneity among patients with similar diagnosis, as well as intratumor heterogeneity, generates difficulties in terms of targeted therapy. Furthermore, late diagnosis represents an ongoing issue, significantly reducing the response to therapy and, inevitably, the overall survival. The role of non-coding RNAs in bladder cancer emerged in the last decade, revealing that microRNAs (miRNAs) may act as tumor suppressor genes, respectively oncogenes, but also as biomarkers for early diagnosis. Regarding other types of non-coding RNAs, especially long non-coding RNAs (lncRNAs) which are extensively reviewed in this article, their exact roles in tumorigenesis are—for the time being—not as evident as in the case of miRNAs, but, still, clearly suggested. Therefore, this review covers the non-coding RNA expression profile of bladder cancer patients and their validated target genes in bladder cancer cell lines, with repercussions on processes such as proliferation, invasiveness, apoptosis, cell cycle arrest, and other molecular pathways which are specific for the malignant transformation of cells.

## 1. Introduction

Bladder cancer is one of the most common malignancies worldwide [1]. It includes two major types: non-muscle invasive bladder cancer (NMIBC) and muscle-invasive bladder cancer (MIBC). NMIBC treatment is represented by chemo- and/or immune-therapy, and requires constant monitoring to prevent the recurrence and evolution to muscle-invasive disease. Bladder cancer represents a major concern due to its frequent relapses and poor clinical outcome once the tumors progress to muscle-invasive disease [2]. Patients with MIBC have limited survival rates due to the aggressive nature of the tumor and its characteristic resistance to chemo- and radiotherapy [3]. Furthermore, in patients with MIBC, the risk of developing lymph node metastases increases and chemotherapy becomes inefficient [4]. Up to date, the prognosis and treatment for patients with bladder cancer are based on their clinical and pathological parameters. The main drawback of these methods is that, even in some cases when tumors present similar histology, they respond differently to the same treatment [5,6]. Therefore, conventional methods and molecular biomarkers should be coupled in order to improve the clinical care of these patients, as well as their outcomes [7].

The metabolic transformations that trigger the switch from a normal cell to malignant bladder cancer cells are diverse and include various classes of molecules that act synergistically in extensive networks that affect normal cell behavior. These changes include the glucose metabolism, Phosphatidylinositol-4,5-bisphosphate 3-kinase/Protein Kinase B/mTOR (PI3K/AKT/mTOR) pathway, glycogen metabolism, and lipid metabolism [8]. The bio-molecular profile for bladder cancer patients is described as having alterations that affect the cellular cycle cyclin-dependent kinase Inhibitor 2A(CDKN2A)/CDK4/CCND1, PI3K/AKT/mTOR), but also survival and growth Receptor tyrosine kinases/RAS (RTK/RAS) pathways, with FGFR3 mutation incidence higher in NMIBC [9]. All these modifications entail different receptors, transporters, enzymes, but also non-coding RNA molecules. All these players can represent targets for precision medicine therapies.

## 2. Non-Coding RNAs

About 90% of the human genome is transcribed, but only 2% of the total genome-sequence is represented by protein-coding genes, the rest being considered, until recently, ‘transcriptional noise’ [10]. With the progress of high-throughput DNA sequencing and array-based technologies, studies revealed that the human transcriptome produces various classes of functional non-coding RNAs which act as regulators of gene expression and play important parts in human diseases [11]. There are two major classes of non-coding RNAs: small non-coding RNA and long non-coding RNA (lncRNA). The mature forms of these non-coding RNAs are never translated into proteins, but they act as regulators of gene expression. The mechanism of action assumes the complementary binding of the RNA molecules to the single stranded mRNA, thus blocking the translation into proteins of certain genes, or leading to the degradation of the mRNA, a process that has the same outcome [12]. Besides, long non-coding RNAs (lncRNAs) are known being also involved in the epigenetic and post- transcriptional regulation of various genes whose alterations lead to a malignant phenotype [13].

### 2.1. Micro-RNAs

Micro-RNAs (miRNAs) are an example of a small non-coding RNA subclass that has been intensively studied in the past few years. With sizes of approximately 22 nucleotides, these miRNAs act as post-transcriptional regulators of gene expression [14]. In addition, there is an increasing number of publications regarding the role of these non-coding RNAs as tumor suppressor genes or as oncogenes [15,16]. Concerning their role in malignant cells, miRNAs are proficient in altering highly regulated mRNA networks [17]. Abnormally expressed miRNAs in bladder cancer are of crucial importance, as they can represent valuable indications of the molecular mechanisms that underlie the development, progression, and metastasis in this type of malignancy. Besides, their small size and their ability to be extravasated from cells via exosomes prevent them from being digested by RNAses in the blood flow, enabling them to move freely within biological fluids (cell-free miRNAs) [18,19]. Therefore, miRNAs represent a category of interest due to their potential significance as biomarkers for the diagnosis/prognosis of patients with bladder cancer, by means of using minimally invasive methods [20].

There is an increasing number of studies concerning this issue which have reported downregulated [21,22,23,24,25] or upregulated [22,26,27] miRNAs in bladder cancer. These aberrantly expressed miRNAs in bladder cancer may reveal specific signatures, such as the miR-195/497 cluster, which acts as a tumor suppressor by modulating the expression of several genes, including *BIRC5* [21]—a gene associated with inhibition of apoptosis [28]. In bladder urothelial carcinoma, miR-182 and miR-183 were reported to be upregulated and miR-143 downregulated, when compared with normal urothelial tissue [22], results that confirm the published work of other researchers [26,27]. The downregulation of miR-8 family members in MIBC compared with NMIBC was previously reported [29] and confirmed [24]. The family members include miR-141, miR-200a, miR-200b, miR-200c, and miR-429, and they exert their function of inhibiting the epithelial-to-mesenchymal transition (EMT) by complementary binding to the Zinc finger E-box-binding homeobox 1/2 genes (*ZEB1/2*) [30].

**Figure 1:**
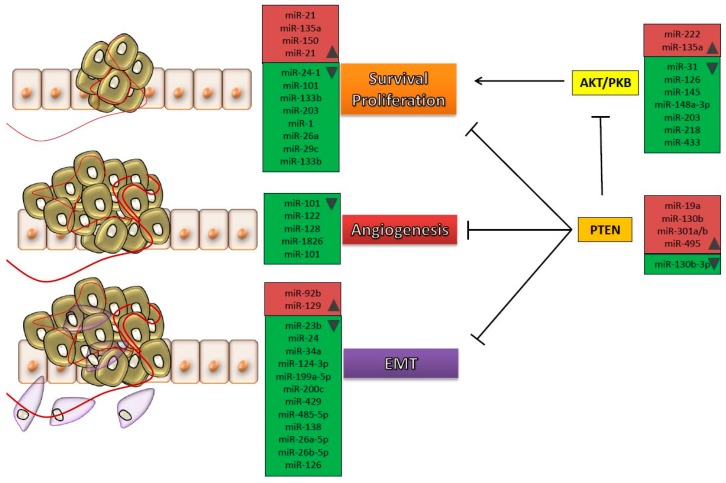
Processes affected by different microRNAs (miRNAs) that were found to be dysregulated in patients with bladder cancer. Cancer cells are subjected to different molecular mechanisms in order to proliferate and invade secondary sites. The main hallmarks consist in survival and proliferation, development of new vascular networks and acquisition of invasive characteristics within epithelial to mesenchymal transition (EMT). All these processes are controlled by aberrantly expressed miRNAs, throughout the progression of tumor cells towards metastasis. In red are represented the upregulated microRNAs and in green those downregulated in bladder cancer; the arrow represents promotion of a process, and the T bar represents suppression of a process.

The crucial importance of these miRNAs in bladder cancer is revealed by the fact that they target critical genes connected to pathways that regulate proliferation [31], invasiveness [32,33,34,35], apoptosis [36,37], resistance to therapy [38], and other features that define the malignant transformation. These unnatural aspects arise from the disruption of different pathways, such as EMT, PI3K/AKT signaling, oxidative stress, Wnt signaling pathways etc. (Figure 1). It was previously reported that microRNAs are useful biomarkers for diagnostic and prognostic, for differentiating tumor tissue from normal tissue, and for stratification of patients with the same expression pattern [39]. Table 1 presents the studies reporting the validated target genes of various miRNAs that were found to be dysregulated in bladder cancer patients and in various cell lines. The validation of the target genes for each miRNA in Table 1 was assessed on bladder cancer cell lines. As expected, the aberrantly expressed miRNAs have been observed to disturb metabolic pathways associated with malignant transformation.

### 2.2. Long Non-Coding RNAs

An important difference between lncRNAs and miRNAs lies in their size, with lncRNAs having more than 200 nucleotides. These types of non-coding RNAs share different characteristics with messenger RNAs, as they comprise several exons. Also, they suffer post-transcriptional modifications, such as splicing and 5′ capping [131]. The importance of these molecules resides in their action as regulators of gene expression. LncRNAs may function by repressing the translation of nearby (*cis*) or faraway (*trans*) genes, by histone modifications or by interfering with the regulatory mechanisms of miRNAs [13,132,133]. The use of lncRNAs as molecular biomarkers emerged with the characterization of the long noncoding RNA PCA3 (prostate cancer antigen 3) and the finding of its upregulated expression in prostate cancer patients [134].

Like other non-coding RNAs, these lncRNA transcripts were related to a wide range of cancer types, including bladder cancer. Alterations in the expression level of these transcripts were proved to modulate an important number of cellular functions, starting with cell proliferation or differentiation, resistance to apoptosis, activation of drug resistance mechanisms or even activation of proangiogenic factors, that eventually contribute to increased invasion and metastatic capacity of tumor cells [135]. Regarding their role in bladder cancer, these non-coding RNAs gained interest in recent years, when the number of studies increased considerably. Table 2 presents the studies published since 2010 that evaluated the expression of different lncRNAs and their putative activity as biomarkers in different types of bladder cancer.

The implication of lncRNAs in bladder cancer tumorigenesis is also reflected in the transcribed ultraconserved regions of the genome, which represent a new type of long noncoding RNAs. It was reported that these transcribed ultraconserved regions act by complementary binding to the sequence of a gene [136].

**Figure 2:**
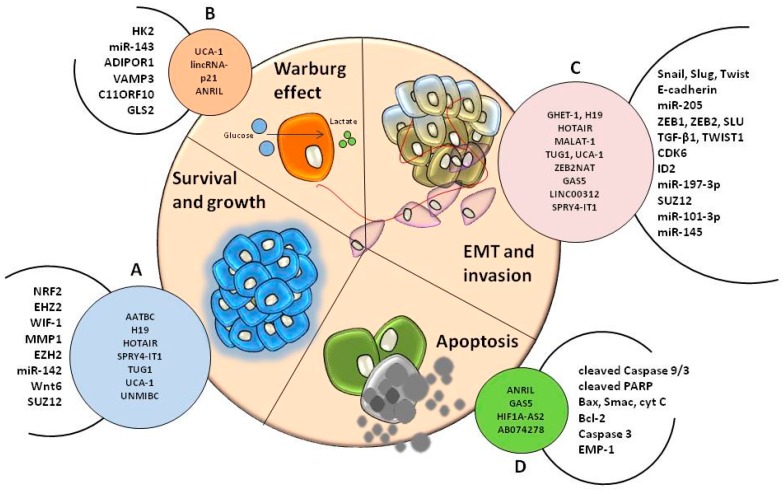
The involvement of lncRNAs in different processes associated with the hallmarks of cancer in bladder malignancies. LncRNAs support the survival and growth of cancer cells (**A**) through interaction with specific target genes and modulation of cell metabolism (**B**). Once the tumor is formed, malignant cells are influenced by non-coding sequences in order to switch towards migratory mesenchymal cells (**C**) within EMT. Apoptosis is also influenced by lncRNAs that are able to target specific genes involved in programmed cell death (**D**) and support the progression of carcinogenesis.

LncRNAs interact with different mRNAs and/or other non-coding RNAs, thus modulating the expression of these transcripts and influencing pathways and processes that lead to the malignant transformation of normal cells into bladder cancer cells. The action of lncRNAs towards chromatin has the capacity to modulate the expression of genes localized near its genomic region (*cis*) or at considerable distance from it (*trans*) [131]. Many studies investigated the association of different lncRNAs with mRNAs of genes which are pivotal in different pathways related to cancer. Table 3 lists the studies that evaluated these associations in bladder cancer cell lines. As it can be noted in Table 3, the interactions between lncRNAs and mRNA/miRNA influence the EMT pathway. The HOTAIR/*ZEB1* interaction affects the EMT in bladder cancer cell lines, HOTAIR being known as a lncRNA associated with tumorigenesis, regulating invasion and cell migration [151]. Likewise, MALAT-1*/E-cadherin* [159] and UCA-1*/ZEB1/2* [184] represent connections that contribute to invasion and metastasis, and were validated in bladder cancer cell lines in a TGF-β-dependent manner [159], or through the hsa-miR-145/ZEB1/2/FSCN1 pathway [184]. The Warburg effect is also depicted in the interactions of UCA-1/miR-143 that affects glycolysis via the mTOR-STAT3 pathway [185]. The way that lncRNAs influence the malignant transformation—disturbing processes as survival, apoptosis, growth, invasion, and Warburg effect—is summarized in Figure 2.

### 2.3. Cell Free microRNAs

There are many processes associated with carcinogenesis that are specific for the tumor stage, grade, aggressiveness, and even for its capacity to metastasize. Some of these abnormalities may also be reflected in specific patterns of miRNA expression in the malignant tissue [189,190]. Conceivably, these modifications may be identified in biofluids, making these miRNAs suitable for their use as minimally invasive biomarkers for cancer detection [191]. These circulating miRNAs can be identified in biofluids in association with ribonucleoprotein complexes, such as Argonaute proteins, or carried by particular extracellular vesicles, including exosomes or other microvesicles [192,193]. There is evidence that indicates the contribution of miRNAs in tumor development, as their release from metastatic cells increases in the blood stream [194]. In bladder cancer, there are various studies that use miRNA expression patterns as biomarkers with prognostic significance, but also for differentiating between the NMIBC and MIBC subtypes [195,196,197,198,199]. Therefore, evaluating the expression of several miRNAs in serum samples, a functional panel of diagnosis biomarkers for MIBC was established, including the aberrantly expressed miR-422a-3, miR-486-3p, miR-103a-3p, and miR-27a-3p. Besides, these miRNAs represent valuable predictors of outcome [195]. At the same time, circulating miRNA expression profiles can represent a signature for bladder cancer, as it was identified a 6-microRNA panel (miR-152, miR-148b-3p, miR-3187-3p, miR-15b-5p, miR-27a-3p and miR-30a-5p) that could be useful for the diagnosis of bladder cancer [196]. In plasma samples, overexpression of miR-205 can represent a biomarker for the early detection of bladder cancer and appears to be associated with poor prognosis [197]. Another study explored the use of urinary miRNAs in bladder cancer, revealing that downregulation of miR-125b in urine supernatant can discriminate between patients with bladder cancer and healthy volunteers [199]. Fendler and colleagues (2016) published a comprehensive table that encompasses studies that assessed miRNAs as potential biomarkers in the blood and urine of patients with bladder cancer [200].

### 2.4. Mitochondrial miRNAs (mitoMiRs)

The mitochondrion is an organelle with crucial importance in several cellular processes, such as metabolic pathways, fatty acid oxidation, aging, apoptosis, and autophagy [201,202]. Regarding mitochondrial function, it is regulated by proteins encoded in the mitochondrial and nuclear genome [203]. Studies show that, in human cell lines, the mitochondrial transcriptome comprises also non-coding RNAs, including miRNA [204]. Therefore, there were indications that the processing of pre-miRNA molecules may take place in the mitochondria, producing mature miRNAs able to target mitochondrial transcripts, or which are transported back to the cytosol, where they can modulate the expression of the nuclear transcripts. These miRNAs appear to be confined, and in association with the mitochondria [205].

The presence of pre-miRNAs and mature miRNAs inside the mitochondria was reported by various studies [206,207] and demonstrated through in situ hybridization for pre-mir-302a, pre-let-7b and mir-365 [208]. One of the mechanisms that change a normal cell into a malignant one is highly connected to the Warburg effect [209] and is driven by multiple factors, including the actions of various miRNAs [210,211]. The Warburg effect intervenes under anaerobic conditions, and the final product of glycolysis, pyruvate, enters the Krebs cycle and is transformed into lactate [210]. One example refers to the indirect action of miR-155 in a miR-143 dependent manner, resulting in the upregulation of hexokinase 2 (HK2)—an enzyme which influences the energy metabolism process demonstrated in breast cancer cells. Precisely, CCAAT/EBPβ is a transcriptional activator for miR-143, which acts as a negative regulator of the *HK2* gene. Upregulation of the *HK2* gene happens as a consequence of miR-155 repressing miR-143 [212,213]. Furthermore, the tumor suppressant activity of miR-126 via regulation of mitochondrial function and metabolism in cancer cells was assessed in malignant mesothelioma [214].

Up to date, there are no published studies regarding the role of mitochondrial miRNAs in bladder cancer. However, it was reported that lncRNA UCA1 derived from the mitochondrial transcriptome might contribute to the protection of the mitochondrial function, being actively involved in the reduction of reactive oxygen species (ROS) by targeting miR-16 in nasopharyngeal carcinoma tissues [215].

### 2.5. The Role of Non-Coding RNAs in Warburg Effect

Metabolism is a key component of the tumor microenvironment. Therefore, the role of aerobic glycolysis—also called Warburg effect—has been extensively studied. In spite of the fact that the metabolic pathways altered in cancer are just beginning to be understood, there are already some non-coding RNAs that have been related to bladder cancer [216]. The most relevant miRNAs that regulate this effect are those that target genes such as cMyc, HIF-1 and P53, active in tumor metabolism. There is the case of a lentiviral sponge for miRNA-21 that has the capacity to reduce glycolysis in bladder cancer T24 cells, via regulation of the PTEN/PI3K/AKT/mTOR axis. It was demonstrated that miR-195-5p inhibits the glucose uptake and proliferation of T24 bladder cancer cells, via modulation of glucose transporters [217].

Presently, several therapeutic combinations are tested using miRNA mimics or inhibitors targeting the Warburg effect, for example the combination of synthetic miR-145 with siRNA for *PTBP1* [218].

The biochemical aspects of the Warburg effect clearly demonstrate their relation to cell growth and proliferation. ANRIL is a well-known lncRNA in a wide range of malignant pathologies, including bladder cancer. Recently, these ncRNAs were demonstrated to regulate the expression of genes involved in glucose and fatty acid metabolism, such as *ADIPOR1*, *VAMP3* and *C11ORF10* [219]. The lncRNA named urothelial cancer associated 1 (UCA1) has an oncogenic role considered to be related with disease progression, and appears to be a promising prognostic marker. UCA1 was associated with promoting glucose consumption and lactate production in bladder cancer cells via a mechanism regulated by HK2 mRNA expression through the mTOR–STAT3 pathway [185]. It was reported that lincRNA-p21 has an important role in regulating Warburg effect via HIF-1α pathway [220].

## 3. Challenges and Future Perspective

The advance of medical technologies allowed the progress in oncological research, leading to a better understanding of the molecular aspects underlying the initiation and development of threatening malignancies. In this context, researchers found that specific untranslated parts of the genome, specifically miRNAs and lncRNAs, are able to influence, at transcriptomic and translational level, the expression of their target genes. This regulation mechanism based on complementary interactions is finely controlled in homeostatic states; however, the balance is lost in malignant scenarios where oncogenic miRNAs and lncRNAs are usually overexpressed, and the tumor suppressor ones are inhibited. This allowed researchers to investigate the extended role of non-coding RNAs in cancer development and progression, and their essential part in precision medicine applications. This review illustrates that both miRNAs and lncRNAs regulate the expression of their target genes, influencing pathways that control the metastatic process, migration, invasion, cell cycle arrest, and cell proliferation of bladder cancer cells. Besides, the presence and the action of particular cells within the tumor microenvironment complete the perturbations that cause tumor heterogeneity in bladder cancer patients. The data accumulating in this field can offer a perspective regarding the molecular profile of patients with bladder cancer in order to use these non-coding RNAs as therapeutic agents designed to target crucial genes dysregulated in bladder cancer, as applications in precision medicine. In this regard, ncRNA expression profiles from tissue, but also from body fluids, can become valuable diagnosis and prognosis markers, where the expression of the target sequences is markedly different between healthy controls and malignant groups. Moreover, specific sets of non-coding RNAs can be used to differentiate between cancer stages and even cancer subtypes. In terms of novel therapeutic alternatives, inhibition of oncogenic miRNAs, and more recently lncRNAs, or overexpression of tumor suppressor ones is also starting to make its way into the clinic. Due to the capacity of ncRNAs to target multiple genes and influence extended signaling networks, experimental modulation of these sequences is opening new alternatives for precision medicine, where patients can be treated according to their individual genetic profile. To conclude, this review gathers the studies that assessed the aberrant expression of miRNAs and lncRNAs, in order to offer a detailed perspective about the non-coding profile in bladder cancer, a profile with valuable meaning in the context of new diagnosis and prognosis tools, but also alternative therapeutic strategies.

## Figures and Tables

**Table 1:** Studies reporting the validated target genes for miRNAs in bladder cancer cells.

miRNA	Expression	Target Genes	Pathway	Reference
miR-9	↑	*CBX7*		[40]
miR-9	↑	*LASS2*		[41]
miR-19a	↑	*PTEN*	AKT/PKB signaling	[42]
miR-21	↑	*BCL-2*	PI3K-AKT	[36]
miR-24-3p	↑	*DEDD*		[43]
miR-92	↑	*GSK3β*	Wnt/c-myc/MMP7 signaling	[44]
miR-92b	↑	*DAB2IP*	EMT	[32]
miR-96	↑	*CDKN1A*		[45]
miR-129	↑	*SOX4*		[25]
miR-130b	↑	*PTEN*	PI3K/AKT signaling	[46]
miR-135a	↑	*PHLPP2*, *FOXO1*	AKT signaling	[47]
miR-137	↑	*PAQR3*		[48]
miR-138-5p	↑	*BIRC5*		[49]
miR-150	↑	*PDCD4*		[50]
miR-155	↑	*DMTF1*		[51]
miR-182-5p	↑	*RECK*, *Smad4*		[52]
miR-193a-3p	↑	*LOXL4*, *SRSF2*	Oxidative Stress	[53]
miR-200	↑	*ERRFI-1*	EGFR inhibitor resistance	[54]
miR-222	↑	*PPP2R2A*	PPP2R2A/AKT/mTOR signaling	[55]
miR-301a/b	↑	*PTEN*	PI3K/AKT signaling	[46]
miR-495	↑	*PTEN*		[56]
miR-1	↓	*LASP1*		[57]
miR-1	↓	*TAGLN2*		[58]
miR-1	↓	*SRSF9*		[59]
miR-1	↓	*PTMA*, *PNP*		[60]
miR-1	↓	*UCA1*		[61]
miR-23b	↓	*Zeb-1*	EMT	[33]
miR-24	↓	*CARMA3*	EMT	[62]
miR-24-1	↓	*FOXM1*		[63]
miR-26a	↓	*HMGA1*		[64]
miR-26a-5p	↓	*PLOD2*		[65]
miR-26b-5p	↓	*PLOD2*		[65]
miR-27a	↓	*SLC7A11*	Glutathione biosynthesis	[66]
miR-27a	↓	*RUNX-1*		[67]
miR-29c	↓	*BCL-2*, *MCL1*		[68]
miR-29c	↓	*CDK6*	G1 phase arrest	[69]
miR-31	↓	*ITGA5*	AKT and ERK	[70]
miR-34a	↓	*Cdk-6*, *SIRT-1*		[71]
miR-34a	↓	*CD44*	EMT	[72]
miR-34a	↓	*HNF4G*		[73]
miR-99a	↓	*FGFR3*		[74]
miR-99a	↓	*FGFR3*		[75]
miR-100	↓	*FGFR3*		[74]
miR-100	↓	*mTOR*		[76]
miR-101	↓	*COX-2*		[77]
miR-101	↓	*VEGF-C*		[78]
miR-101	↓	*c-FOS*		[79]
miR-106a	↓	*cyclin D1/CDK6*		[80]
miR-122	↓	*VEGFC*	mTOR and AKT	[81]
miR-124	↓	*Cdk4*		[82]
miR-124	↓	*UHRF1*		[83]
miR-124-3p	↓	*ROCK1*	EMT	[34]
miR-125b	↓	*E2F3*	E2F3–Cyclin A2 signaling	[84]
miR-125b	↓	*MMP13*		[85]
miR-125b	↓	*SphK1*	G1 phase arrest	[86]
miR-126	↓	*ADAM9*		[87]
miR-126	↓	*PIK3R2*	PI3K/AKT signaling	[88]
miR-128	↓	*VEGF-C*		[89]
miR-1280	↓	*ROCK1*		[90]
miR-130b-3p	↓	*PTEN*	PI3K and integrin β1/FAK signaling	[91]
miR-133a	↓	*LASP1*		[57]
miR-133a	↓	*TAGLN2*		[58]
miR-133a	↓	*PTMA*, *PNP*		[60]
miR-133a/b	↓	*EGFR*, *MMP-2*		[92]
miR-133b	↓	*Bcl-w*, *AKT1*		[37]
miR-138	↓	*ZEB2*		[93]
miR-139-3p/5p	↓	*MMP11*		[94]
miR-143	↓	*IGF-1R*		[95]
miR-143/145 cluster	↓	*PAI-1*		[96]
miR-144	↓	*EZH2*	Wnt signaling/EZH2/Nkd1	[97]
miR-144-5p	↓	*CCNE1/2*, *CDC25A*		[31]
miR-145	↓	*FSCN1*		[98]
miR-145	↓	*SOCS7*	PI3K/AKT signaling	[99]
miR-145	↓	*PAK1*		[100]
miR-145	↓	*IGF-IR*		[101]
miR-145-3p/5p	↓	*UHRF1*		[102]
miR-146-3p	↓	*PTTG1*		[103]
miR-148a-3p	↓	*ERBB3*, *DNMT1, AKT2*		[104]
miR-1826	↓	*VEGFC*, *CTNNB1, MAP2K1*	MAPK-ERK signal transduction	[105]
miR-186	↓	*NSBP1*		[106]
miR-193-3p	↓	*HOXC9*	DNA damage response and oxidative stress	[107]
miR-194	↓	*RAP2B*		[108]
miR-195	↓	*Cdk4*	G1-phase arrest	[109]
miR-195	↓	*Cdc42*	Cdc42/STAT3 signaling	[110]
miR-195/497 cluster	↓	*BIRC5*, *WNT7A*		[21]
miR-199-3p/5p	↓	*ITGA3*		[111]
miR-199a-3p/5p	↓	*ITGA3*		[111]
miR-199a-5p	↓	*MLK3*	MLK3/IκB/NF-κB	[112]
miR-199a-5p	↓	*CCR7*	EMT	[35]
miR-200c	↓	*BMI-1*, *E2F3*	EMT	[113]
miR-203	↓	*bcl-w*		[114]
miR-203	↓	*AKT2*, *Src*	PI3K/AKT signaling	[115]
miR-205	↓	*CCNJ*		[116]
miR-206	↓	*YRDC*		[104]
miR-214	↓	*PDRG1*		[117]
miR-218	↓	*LASP1*		[57]
miR-218	↓	*BMI-1*	PI3K/AKT signaling	[118]
miR-218	↓	*Glut1*		[119]
miR-320c	↓	*Cdk6*	G1-phase arrest	[120]
miR-320s	↓	*ITGB3*		[121]
miR-335	↓	*ROCK1*		[122]
miR-335	↓	*MAPK1*		[38]
miR-409-3p	↓	*c-Met*		[123]
miR-429	↓	*E-cad*, *ZEB1/2, β-catenin*	EMT	[124]
miR-430	↓	*CXCR3*		[125]
miR-433	↓	*CREB*, *c-Met*	c-Met/AKT/GSK-3β/Snail signaling, EMT	[126]
miR-451	↓	*c-Myc*		[127]
miR-485-5p	↓	*HMGA2*	EMT	[128]
miR-490-5p	↓	*c-FOS*	G1-phase arrest	[129]
miR-490-5p	↓	*c-FOS*		[130]

AKT/PKB, Protein kinase B; PI3K-AKT, Phosphatidylinositol-4,5-bisphosphate 3-kinase-Protein kinase B; EMT, epithelial-mesenchymal transition; EGFR, Epidermal growth factor receptor; PPP2R2A, Serine/threonine-protein phosphatase 2A 55 kDa regulatory subunit B alpha isoform; ↑, upregulated expression; ↓, downregulated expression.

**Table 2:** Studies assessing the expression of different long non-coding RNAs (lncRNAs) in bladder cancer patients.

lncRNA	Tumor Type	Study	Expression	Reference
AATBC	Bladder cancer	90 patients 90 adjacent normal tissue	↑ 54 cases ↓ 36 cases	[137]
ANRIL	Bladder cancer	51 patients 51 adjacent non-tumor tissues	↑	[138]
BANCR	Bladder cancer	54 patients 54matched adjacent normal tissues	↓	[139]
GAS5	Urothelial carcinoma	28 patients 28 adjacent normal mucosa	↓	[140]
GAS5	Transitional cell carcinoma	82 patients 37 normal bladder tissues	↓	[141]
GHET-1	Bladder cancer	80 patients 80 adjacent normal tissues	↑	[142]
H19	Transitional cell carcinoma	39 patients	↑	[143]
H19	Urothelial carcinoma	41 patients 41 adjacent normal mucosa	↑	[144]
H19	Urothelial carcinoma	24 patients 24 adjacent normal control tissues	↑	[145]
H19	Bladder cancer	40 patients 19 adjacent normal tissues	↑	[146]
HIF1A-AS2	Bladder cancer	44 patients 44 matched normal peritumoral tissues	↑	[147]
HOTAIR	Urothelial carcinoma	110 patients 110 matched normal tissues	↑ 90 cases ↓ 20 cases	[148]
HOTAIR	Urothelial carcinoma	Set 1: 19 patients, 10 normal bladder tissues Set 2: 108 patients, 7 normal bladder tissues	Set 1: ↑ 9 cases Set 2: No significant differences	[149]
HOTAIR	Transitional cell carcinoma	35 patients 16 normal bladder transitional cell	↑	[150]
HOTAIR MALAT-1	Bladder cancer	10 patients 10 distal normal tissue	↑ ↑	[151]
HOTAIR	NMIBC	64 patients 64 distant normal mucosa	↑	[152]
LINC00312	Bladder cancer	110 patients	↓	[153]
Linc-UBC1	Bladder cancer	102 patients 102 adjacent normal mucosa	↑ 60 cases ↓ 42 cases	[154]
LOC572558	Bladder cancer	24 patients 24 matched normal bladder tissue	↓	[155]
MALAT-1	Bladder cancer	22 patients 22 matched normal tissues	↑	[156]
MALAT-1	Urothelial carcinoma	36 patients 36 matched histologically normal urothelium	↑	[157]
MALAT-1	Urothelial carcinoma	27 patients 27 adjacent histologically normal tissues	↑	[158]
MALAT-1	Urothelial carcinoma	95 patients 95 matched normal tissues	↑	[159]
MDC1-AS	Bladder cancer	32 patients 32 adjacent normal tissue	↓	[160]
MEG3	Urothelial carcinoma	31 patients 31 adjacent benign tissues	↓	[161]
MIR31HG	Bladder cancer	55 patients 55 matched non-tumor bladder tissues	↓	[162]
MIR31HG	Bladder cancer	55 patients 55 adjacent normal tissue	↓	[162]
n336928	Bladder cancer	95 patients 95 adjacent non-tumor tissues	↑	[163]
ncRAN	Transitional cell carcinoma	40 patients 40 paired adjacent noncancerous tissues	↑	[164]
NEAT1	Bladder cancer	65 patients 65 adjacent normal tissue	↑ 48 cases	[165]
PCAT-1	Urothelial carcinoma	36 patients 36 matched normal tissues	↑	[166]
PVT1	Bladder cancer	32 patients 32 matched para-cancer tissues	↑ 20 cases	[167]
SChLAP1	Urothelial carcinoma	36 patients 36 paired normal bladder tissue	↑	[168]
SPRY4-IT1	Urothelial carcinoma	68 patients 68 adjacent non-tumor tissues	↑	[169]
SPRY4-IT1	Bladder cancer	60 patients 60 matched normal adjacent tissue	↑	[170]
TUG1	Urothelial carcinoma	44 patients 44 matched histologically normal urothelium	↑	[171]
TUG1	Bladder cancer	36 patients 36 adjacent normal tissue	↑	[172]
TUG1	Bladder cancer	54 patients	↑	[173]
TUG1	Bladder cancer	47 patients 47 adjacent non-tumor bladder tissues	↑	[174]
UCA1	Urothelial carcinoma	34 patients (Cisplatin-based chemotherapy)	↑	[175]
UCA1	Urothelial carcinoma	20 patients 20 matched normal tissues	↑ 17 cases ↓ 3 cases	[176]
UCA1	Urothelial carcinoma	25 patients 25 matched normal tissues	↑	[61]
UCA1	Transitional cell carcinoma	117 patients 74 non bladder cancer patients	↑	[177]
UCA1	Bladder cancer	94 patients urine samples from patients with benign disease (56) or healthy volunteers (60)	↑	[178]
UCA1	Bladder cancer	184 patients (139 malignant disease+45 benign disease) 36 healthy volunteers	↑	[179]
UCA1	Bladder cancer	35 patients 6 normal bladder tissues 10 adjacent normal tissues	↑	[180]
UCA1a (CUDR)	Transitional cell carcinoma	8 patients 8 adjacent normal mucosa	↑	[181]
uc.8+ (ultraconserved RNA 8+)	Bladder cancer	24 patients 17 normal bladder epithelium	↑	[136]
UNMIBC	NMIBC	75 patients 75 adjacent normal mucosa	↑ 45 cases	[182]
ZEB2NAT	Urothelial carcinoma	30 patients 30 adjacent normal tissue	↑	[183]

AATBC, Apoptosis Associated Transcript In Bladder Cancer; ANRIL, Antisense Noncoding RNA in the INK4 Locus; BANCR, BRAF activated non-coding RNA; GAS5, Growth arrest-specific 5; GHET-1, gastric carcinoma high expressed transcript 1, HIF1A-AS2, hypoxia inducible factor 1 alpha-antisense RNA 2; HOTAIR, HOX transcript antisense RNA; LINC00312, long intergenic noncoding RNA; linc-UBC1, long intergenic RNA- Up-regulated in bladder cancer 1; MALAT-1, Metastasis Associated Lung Adenocarcinoma Transcript 1; MDC1-AS, mediator of DNA damage checkpoint protein 1antisense RNA; MEG3, maternally expressed 3; MIR31HG, host gene of miR-31; NEAT1, Nuclear Enriched Abundant Transcript 1; PCAT1, prostate cancer associated transcript 1; SChLAP1, SWI/SNF Complex Antagonist Associated With Prostate Cancer 1; SPRY4-IT1, SPRY4 intronic transcript 1; TUG1, taurine upregulated gene 1; UCA1, urothelial cancer associated 1; UNMIBC, up-regulated in nonmuscle invasive bladder cancer; ↑, upregulated expression; ↓, downregulated expression

**Table 3:** List of studies evaluating the expression of different lncRNAs and their putative target genes in bladder cancer.

lncRNA	Association with Genes	Pathway	Reference
AATBC	↑ *NRF2*	JNK signaling	[137]
AB074278	↓ *EMP-1*		[186]
ANRIL	↓ *cleaved Caspase 9/3*, *cleaved PARP*, *Bax*, *Smac*, *cyt C* ↑ *Bcl-2*	Intrinsic pathway	[138]
GAS5	↓ *CDK6*		[140]
GAS5	↓ *CCL1*		[187]
GAS5	↓ *Bcl-2*		[141]
GHET-1	↓ *Snail*, *Slug*, *Twist*, *ZEB1*	EMT	[142]
H19	*EHZ2* ↓ *E-cadherin*	Wnt/β-catenin	[144]
H19	↑ *ID2*		[145]
HIF1A-AS2	↑ *caspase 3*		[147]
HOTAIR	↓ *WIF-1*	Wnt/β-catenin signaling	[148]
HOTAIR	↓ *miR-205*		[152]
HOTAIR	↑ *SNAI1*, *ZEB1*, *TWIST1*, *MMP1*	EMT	[151]
LINC00312	↓ *miR-197-3p*		[153]
Linc-UBC1	*PRC complex*		[154]
LOC572558	↑ *AKT*, *MDM2*, *p53*	AKT-MDM2-p53 signaling axis	[155]
MALAT-1	↓ *ZEB1*, *ZEB2*, *SLU*, *E-cadherin*	Wnt/β-catenin	[156]
MALAT-1	↓ *E-cadherin* *SUZ12*	EMT	[159]
SPRY4-IT1	↓ *miR-101-3p* ↑ *EZH2*	EMT EZH2 pathway	[170]
SPRY4-IT1	↑ *miR-101-3p* ↓ *EZH2*		[170]
TUG1	↑ *ZEB2* ↓ *miR-142*	Wnt/β-catenin pathway	[172]
TUG1	↓ *miR-145*	EMT	[173]
UCA-1	↑ *HK2* ↓ *miR-143*	Glycolysis mTOR–STAT3 pathway	[185]
UCA-1	↑ *CREB*	PI3-K/AKT	[188]
UCA-1	↑ *Wnt6*	Wnt signaling	[175]
UCA-1	↓ *BRG1*		[176]
UCA-1	↑ *GLS2*	Cell redox state Glutaminolysis	[180]
UCA-1	↑ *ZEB1/2* ↓ *miR-145*	EMT miR-145–ZEB1/2–FSCN1	[184]
UNMIBC	*EZH2*, *SUZ12*		[182]
ZEB2NAT	↑ *TGF-β1*, *ZEB2*	EMT	[183]

## Abbreviations

BC	Bladder cancer
MIBC	Muscle invasive bladder cancer
NMIBC	Non-muscle invasive bladder cancer
mitoMir	Mitochondrial microRNA

## References

[1] Ferlay J., Steliarova-Foucher E., Lortet-Tieulent J., Rosso S., Coebergh J.W., Comber H., Forman D., Bray F. (2013). Cancer incidence and mortality patterns in Europe: Estimates for 40 countries in 2012. Eur. J. Cancer.

[2] Van Rhijn B.W., Burger M., Lotan Y., Solsona E., Stief C.G., Sylvester R.J., Witjes J.A., Zlotta A.R. (2009). Recurrence and progression of disease in non-muscle-invasive bladder cancer: From epidemiology to treatment strategy. Eur. Urol..

[3] Drayton R.M., Catto J.W. (2012). Molecular mechanisms of cisplatin resistance in bladder cancer. Expert Rev. Anticancer Ther..

[4] Carballido E.M., Rosenberg J.E. (2014). Optimal treatment for metastatic bladder cancer. Curr. Oncol. Rep..

[5] Jacobs B.L., Lee C.T., Montie J.E. (2010). Bladder cancer in 2010: How far have we come?. CA Cancer J. Clin..

[6] Roupret M., Hupertan V., Yates D.R., Comperat E., Catto J.W., Meuth M., Lackmichi A., Ricci S., Lacave R., Gattegno B. (2008). A comparison of the performance of microsatellite and methylation urine analysis for predicting the recurrence of urothelial cell carcinoma, and definition of a set of markers by Bayesian network analysis. BJU Int..

[7] Ye F., Wang L., Castillo-Martin M., McBride R., Galsky M.D., Zhu J., Boffetta P., Zhang D.Y., Cordon-Cardo C. (2014). Biomarkers for bladder cancer management: Present and future. Am. J. Clin. Exp. Urol..

[8] Massari F., Ciccarese C., Santoni M., Iacovelli R., Mazzucchelli R., Piva F., Scarpelli M., Berardi R., Tortora G., Lopez-Beltran A. (2016). Metabolic phenotype of bladder cancer. Cancer Treat. Rev..

[9] Cancer Genome Atlas Research Network (2014). Comprehensive molecular characterization of urothelial bladder carcinoma. Nature.

[10] Gibb E.A., Brown C.J., Lam W.L. (2011). The functional role of long non-coding RNA in human carcinomas. Mol. Cancer.

[11] Taft R.J., Pang K.C., Mercer T.R., Dinger M., Mattick J.S. (2010). Non-coding RNAs: Regulators of disease. J. Pathol..

[12] Lewis B.P., Shih I.H., Jones-Rhoades M.W., Bartel D.P., Burge C.B. (2003). Prediction of mammalian microRNA targets. Cell.

[13] Martens-Uzunova E.S., Bottcher R., Croce C.M., Jenster G., Visakorpi T., Calin G.A. (2014). Long noncoding RNA in prostate, bladder, and kidney cancer. Eur. Urol..

[14] Bartel D.P. (2004). MicroRNAs: Genomics, biogenesis, mechanism, and function. Cell.

[15] Farazi T.A., Hoell J.I., Morozov P., Tuschl T. (2013). MicroRNAs in human cancer. Adv. Exp. Med. Biol..

[16] Berindan-Neagoe I., Monroig Pdel C., Pasculli B., Calin G.A. (2014). Micrornaome genome: A treasure for cancer diagnosis and therapy. CA Cancer J. Clin..

[17] Kong Y.W., Ferland-McCollough D., Jackson T.J., Bushell M. (2012). microRNAs in cancer management. Lancet Oncol..

[18] Fevrier B., Raposo G. (2004). Exosomes: Endosomal-derived vesicles shipping extracellular messages. Curr. Opin. Cell Biol..

[19] Braicu C., Tomuleasa C., Monroig P., Cucuianu A., Berindan-Neagoe I., Calin G.A. (2015). Exosomes as divine messengers: Are they the hermes of modern molecular oncology?. Cell Death Differentiation.

[20] Berindan-Neagoe I., Calin G.A. (2014). Molecular pathways: microRNAs, cancer cells, and microenvironment. Clin. Cancer Res..

[21] Itesako T., Seki N., Yoshino H., Chiyomaru T., Yamasaki T., Hidaka H., Yonezawa T., Nohata N., Kinoshita T., Nakagawa M. (2014). The microRNA expression signature of bladder cancer by deep sequencing: The functional significance of the miR-195/497 cluster. PLoS ONE.

[22] Han Y., Chen J., Zhao X., Liang C., Wang Y., Sun L., Jiang Z., Zhang Z., Yang R., Chen J. (2011). MicroRNA expression signatures of bladder cancer revealed by deep sequencing. PLoS ONE.

[23] Canturk K.M., Ozdemir M., Can C., Oner S., Emre R., Aslan H., Cilingir O., Ciftci E., Celayir F.M., Aldemir O. (2014). Investigation of key miRNAs and target genes in bladder cancer using miRNA profiling and bioinformatic tools. Mol. Biol. Rep..

[24] Ratert N., Meyer H.A., Jung M., Lioudmer P., Mollenkopf H.J., Wagner I., Miller K., Kilic E., Erbersdobler A., Weikert S. (2013). miRNA profiling identifies candidate mirnas for bladder cancer diagnosis and clinical outcome. J. Mol. Diagn..

[25] Dyrskjot L., Ostenfeld M.S., Bramsen J.B., Silahtaroglu A.N., Lamy P., Ramanathan R., Fristrup N., Jensen J.L., Andersen C.L., Zieger K. (2009). Genomic profiling of microRNAs in bladder cancer: miR-129 is associated with poor outcome and promotes cell death in vitro. Cancer Res..

[26] Friedman J.M., Liang G., Liu C.C., Wolff E.M., Tsai Y.C., Ye W., Zhou X., Jones P.A. (2009). The putative tumor suppressor microRNA-101 modulates the cancer epigenome by repressing the polycomb group protein EZH2. Cancer Res..

[27] Lin T., Dong W., Huang J., Pan Q., Fan X., Zhang C., Huang L. (2009). MicroRNA-143 as a tumor suppressor for bladder cancer. J. Urol..

[28] Kanwar J.R., Kamalapuram S.K., Kanwar R.K. (2013). Survivin signaling in clinical oncology: A multifaceted dragon. Med. Res. Rev..

[29] Wiklund E.D., Bramsen J.B., Hulf T., Dyrskjot L., Ramanathan R., Hansen T.B., Villadsen S.B., Gao S., Ostenfeld M.S., Borre M. (2011). Coordinated epigenetic repression of the miR-200 family and miR-205 in invasive bladder cancer. Int. J. Cancer.

[30] Park S.M., Gaur A.B., Lengyel E., Peter M.E. (2008). The miR-200 family determines the epithelial phenotype of cancer cells by targeting the E-cadherin repressors ZEB1 and ZEB2. Genes Dev..

[31] Matsushita R., Seki N., Chiyomaru T., Inoguchi S., Ishihara T., Goto Y., Nishikawa R., Mataki H., Tatarano S., Itesako T. (2015). Tumour-suppressive microRNA-144–5p directly targets CCNE1/2 as potential prognostic markers in bladder cancer. Br. J. Cancer.

[32] Huang J., Wang B., Hui K., Zeng J., Fan J., Wang X., Hsieh J.T., He D., Wu K. (2016). miR-92b targets DAB2IP to promote EMT in bladder cancer migration and invasion. Oncol. Rep..

[33] Majid S., Dar A.A., Saini S., Deng G., Chang I., Greene K., Tanaka Y., Dahiya R., Yamamura S. (2013). MicroRNA-23b functions as a tumor suppressor by regulating Zeb1 in bladder cancer. PLoS ONE.

[34] Xu X., Li S., Lin Y., Chen H., Hu Z., Mao Y., Xu X., Wu J., Zhu Y., Zheng X. (2013). MicroRNA-124–3p inhibits cell migration and invasion in bladder cancer cells by targeting ROCK1. J. Trans. Med..

[35] Zhou M., Wang S., Hu L., Liu F., Zhang Q., Zhang D. (2016). miR-199a-5p suppresses human bladder cancer cell metastasis by targeting CCR7. BMC Urol..

[36] Tao J., Lu Q., Wu D., Li P., Xu B., Qing W., Wang M., Zhang Z., Zhang W. (2011). MicroRNA-21 modulates cell proliferation and sensitivity to doxorubicin in bladder cancer cells. Oncol. Rep..

[37] Chen X.N., Wang K.F., Xu Z.Q., Li S.J., Liu Q., Fu D.H., Wang X., Wu B. (2014). MiR-133b regulates bladder cancer cell proliferation and apoptosis by targeting Bcl-w and Akt1. Cancer Cell Int..

[38] Wang X., Wu G., Cao G., Chen X., Huang J., Jiang X., Hou J. (2016). MicroRNA335 inhibits bladder cancer cell growth and migration by targeting mitogenactivated protein kinase 1. Mol. Med. Rep..

[39] Braicu C., Cojocneanu-Petric R., Chira S., Truta A., Floares A., Petrut B., Achimas-Cadariu P., Berindan-Neagoe I. (2015). Clinical and pathological implications of mirna in bladder cancer. Int. J. Nanomed..

[40] Xie D., Shang C., Zhang H., Guo Y., Tong X. (2015). Up-regulation of miR-9 target CBX7 to regulate invasion ability of bladder transitional cell carcinoma. Med. Sci. Monit..

[41] Wang H., Zhang W., Zuo Y., Ding M., Ke C., Yan R., Zhan H., Liu J., Wang J. (2015). miR-9 promotes cell proliferation and inhibits apoptosis by targeting LASS2 in bladder cancer. Tumour Biol..

[42] Feng Y., Liu J., Kang Y., He Y., Liang B., Yang P., Yu Z. (2014). miR-19a acts as an oncogenic microRNA and is up-regulated in bladder cancer. J. Exp. Clin. Cancer Res..

[43] Yu G., Jia Z., Dou Z. (2017). MiR-24–3p regulates bladder cancer cell proliferation, migration, invasion and autophagy by targeting DEDD. Oncol. Rep..

[44] Wang H., Ke C., Ma X., Zhao Q., Yang M., Zhang W., Wang J. (2016). MicroRNA-92 promotes invasion and chemoresistance by targeting GSK3β and activating Wnt signaling in bladder cancer cells. Tumour Biol..

[45] Wu Z., Liu K., Wang Y., Xu Z., Meng J., Gu S. (2015). Upregulation of microRNA-96 and its oncogenic functions by targeting CDKN1A in bladder cancer. Cancer Cell Int..

[46] Egawa H., Jingushi K., Hirono T., Ueda Y., Kitae K., Nakata W., Fujita K., Uemura M., Nonomura N., Tsujikawa K. (2016). The miR-130 family promotes cell migration and invasion in bladder cancer through FAK and Akt phosphorylation by regulating PTEN. Sci. Rep..

[47] Mao X.P., Zhang L.S., Huang B., Zhou S.Y., Liao J., Chen L.W., Qiu S.P., Chen J.X. (2015). Mir-135a enhances cellular proliferation through post-transcriptionally regulating PHLPP2 and FOXO1 in human bladder cancer. J. Trans. Med..

[48] Xiu Y., Liu Z., Xia S., Jin C., Yin H., Zhao W., Wu Q. (2014). MicroRNA-137 upregulation increases bladder cancer cell proliferation and invasion by targeting PAQR3. PLoS ONE.

[49] Yang R., Liu M., Liang H., Guo S., Guo X., Yuan M., Lian H., Yan X., Zhang S., Chen X. (2016). miR-138–5p contributes to cell proliferation and invasion by targeting Survivin in bladder cancer cells. Mol. Cancer.

[50] Lei Y., Hu X., Li B., Peng M., Tong S., Zu X., Wang Z., Qi L., Chen M. (2014). miR-150 modulates cisplatin chemosensitivity and invasiveness of muscle-invasive bladder cancer cells via targeting PDCD4 in vitro. Med. Sci. Monit..

[51] Peng Y., Dong W., Lin T.X., Zhong G.Z., Liao B., Wang B., Gu P., Huang L., Xie Y., Lu F.D. (2015). MicroRNA-155 promotes bladder cancer growth by repressing the tumor suppressor DMTF1. Oncotarget.

[52] Hirata H., Ueno K., Shahryari V., Tanaka Y., Tabatabai Z.L., Hinoda Y., Dahiya R. (2012). Oncogenic miRNA-182–5p targets Smad4 and RECK in human bladder cancer. PLoS ONE.

[53] Deng H., Lv L., Li Y., Zhang C., Meng F., Pu Y., Xiao J., Qian L., Zhao W., Liu Q. (2014). miR-193a-3p regulates the multi-drug resistance of bladder cancer by targeting the LOXL4 gene and the oxidative stress pathway. Mol. Cancer.

[54] Adam L., Zhong M., Choi W., Qi W., Nicoloso M., Arora A., Calin G., Wang H., Siefker-Radtke A., McConkey D. (2009). miR-200 expression regulates epithelial-to-mesenchymal transition in bladder cancer cells and reverses resistance to epidermal growth factor receptor therapy. Clin. Cancer Res..

[55] Zeng L.P., Hu Z.M., Li K., Xia K. (2016). miR-222 attenuates cisplatin-induced cell death by targeting the PPP2R2A/Akt/mTOR Axis in bladder cancer cells. J. Cell. Mol. Med..

[56] Tan M., Mu X., Liu Z., Tao L., Wang J., Ge J., Qiu J. (2017). microRNA-495 promotes bladder cancer cell growth and invasion by targeting phosphatase and tensin homolog. Biochem. Biophys. Res. Commun..

[57] Chiyomaru T., Enokida H., Kawakami K., Tatarano S., Uchida Y., Kawahara K., Nishiyama K., Seki N., Nakagawa M. (2012). Functional role of LASP1 in cell viability and its regulation by microRNAs in bladder cancer. Urol. Oncol..

[58] Yoshino H., Chiyomaru T., Enokida H., Kawakami K., Tatarano S., Nishiyama K., Nohata N., Seki N., Nakagawa M. (2011). The tumour-suppressive function of miR-1 and miR-133a targeting TAGLN2 in bladder cancer. Br. J. Cancer.

[59] Yoshino H., Enokida H., Chiyomaru T., Tatarano S., Hidaka H., Yamasaki T., Gotannda T., Tachiwada T., Nohata N., Yamane T. (2012). Tumor suppressive microRNA-1 mediated novel apoptosis pathways through direct inhibition of splicing factor serine/arginine-rich 9 (SRSF9/SRp30c) in bladder cancer. Biochem. Biophys. Res. Commun..

[60] Yamasaki T., Yoshino H., Enokida H., Hidaka H., Chiyomaru T., Nohata N., Kinoshita T., Fuse M., Seki N., Nakagawa M. (2012). Novel molecular targets regulated by tumor suppressors microRNA-1 and microRNA-133a in bladder cancer. Int. J. Oncol..

[61] Wang T., Yuan J., Feng N., Li Y., Lin Z., Jiang Z., Gui Y. (2014). Hsa-miR-1 downregulates long non-coding RNA urothelial cancer associated 1 in bladder cancer. Tumour Biol..

[62] Zhang S., Zhang C., Liu W., Zheng W., Zhang Y., Wang S., Huang D., Liu X., Bai Z. (2015). MicroRNA-24 upregulation inhibits proliferation, metastasis and induces apoptosis in bladder cancer cells by targeting CARMA3. Int. J. Oncol..

[63] Inoguchi S., Seki N., Chiyomaru T., Ishihara T., Matsushita R., Mataki H., Itesako T., Tatarano S., Yoshino H., Goto Y. (2014). Tumour-suppressive microRNA-24–1 inhibits cancer cell proliferation through targeting FOXM1 in bladder cancer. FEBS Lett..

[64] Lin Y., Chen H., Hu Z., Mao Y., Xu X., Zhu Y., Xu X., Wu J., Li S., Mao Q. (2013). MiR-26a inhibits proliferation and motility in bladder cancer by targeting HMGA1. FEBS Lett..

[65] Miyamoto K., Seki N., Matsushita R., Yonemori M., Yoshino H., Nakagawa M., Enokida H. (2016). Tumour-suppressive miRNA-26a-5p and miR-26b-5p inhibit cell aggressiveness by regulating PLOD2 in bladder cancer. Br. J. Cancer.

[66] Drayton R.M., Dudziec E., Peter S., Bertz S., Hartmann A., Bryant H.E., Catto J.W. (2014). Reduced expression of miRNA-27a modulates cisplatin resistance in bladder cancer by targeting the cystine/glutamate exchanger SLC7A11. Clin. Cancer Res..

[67] Deng Y., Bai H., Hu H. (2015). rs11671784 G/A Variation in miR-27a decreases chemo-sensitivity of bladder cancer by decreasing miR-27a and increasing the target RUNX-1 expression. Biochem. Biophys. Res. Commun..

[68] Xu X.D., Wu X.H., Fan Y.R., Tan B., Quan Z., Luo C.L. (2014). Exosome-derived microRNA-29c induces apoptosis of BIU-87 cells by down regulating BCL-2 and MCL-1. Asian Pac. J. Cancer Prev..

[69] Zhao X., Li J., Huang S., Wan X., Luo H., Wu D. (2015). MiRNA-29c regulates cell growth and invasion by targeting CDK6 in bladder cancer. Am. J. Trans. Res..

[70] Xu T., Qin L., Zhu Z., Wang X., Liu Y., Fan Y., Zhong S., Wang X., Zhang X., Xia L. (2016). MicroRNA-31 functions as a tumor suppressor and increases sensitivity to mitomycin-C in urothelial bladder cancer by targeting integrin α5. Oncotarget.

[71] Vinall R.L., Ripoll A.Z., Wang S., Pan C.X., deVere White R.W. (2012). MiR-34a chemosensitizes bladder cancer cells to cisplatin treatment regardless of p53-Rb pathway status. Int. J. Cancer.

[72] Yu G., Yao W., Xiao W., Li H., Xu H., Lang B. (2014). MicroRNA-34a functions as an anti-metastatic microRNA and suppresses angiogenesis in bladder cancer by directly targeting CD44. J. Exp. Clin. Cancer Res..

[73] Sun H., Tian J., Xian W., Xie T., Yang X. (2015). miR-34a inhibits proliferation and invasion of bladder cancer cells by targeting orphan nuclear receptor HNF4G. Dis. Markers.

[74] Catto J.W., Miah S., Owen H.C., Bryant H., Myers K., Dudziec E., Larre S., Milo M., Rehman I., Rosario D.J. (2009). Distinct microRNA alterations characterize high- and low-grade bladder cancer. Cancer Res..

[75] Wu D., Zhou Y., Pan H., Zhou J., Fan Y., Qu P. (2014). MicroRNA-99a inhibiting cell proliferation, migration and invasion by targeting fibroblast growth factor receptor 3 in bladder cancer. Oncol. Lett..

[76] Xu C., Zeng Q., Xu W., Jiao L., Chen Y., Zhang Z., Wu C., Jin T., Pan A., Wei R. (2013). MiRNA-100 inhibits human bladder urothelial carcinogenesis by directly targeting mTOR. Mol. Cancer Ther..

[77] Bu Q., Fang Y., Cao Y., Chen Q., Liu Y. (2014). Enforced expression of miR-101 enhances cisplatin sensitivity in human bladder cancer cells by modulating the cyclooxygenase-2 pathway. Mol. Med. Rep..

[78] Lei Y., Li B., Tong S., Qi L., Hu X., Cui Y., Li Z., He W., Zu X., Wang Z. (2015). MiR-101 suppresses vascular endothelial growth factor C that inhibits migration and invasion and enhances cisplatin chemosensitivity of bladder cancer cells. PLoS ONE.

[79] Long Y., Wu Z., Yang X., Chen L., Han Z., Zhang Y., Liu J., Liu W., Liu X. (2016). MicroRNA-101 inhibits the proliferation and invasion of bladder cancer cells via targeting c-FOS. Mol. Med. Rep..

[80] Shin S.S., Park S.S., Hwang B., Kim W.T., Choi Y.H., Kim W.J., Moon S.K. (2016). MicroRNA-106a suppresses proliferation, migration, and invasion of bladder cancer cells by modulating MAPK signaling, cell cycle regulators, and Ets-1-mediated MMP-2 expression. Oncol. Rep..

[81] Wang Y., Xing Q.F., Liu X.Q., Guo Z.J., Li C.Y., Sun G. (2016). MiR-122 targets VEGFC in bladder cancer to inhibit tumor growth and angiogenesis. Am. J. Trans. Res..

[82] Zhang T., Wang J., Zhai X., Li H., Li C., Chang J. (2014). MiR-124 retards bladder cancer growth by directly targeting CDK4. Acta Biochim. Biophys. Sinica.

[83] Wang X., Wu Q., Xu B., Wang P., Fan W., Cai Y., Gu X., Meng F. (2015). MiR-124 exerts tumor suppressive functions on the cell proliferation, motility and angiogenesis of bladder cancer by fine-tuning UHRF1. FEBS J..

[84] Huang L., Luo J., Cai Q., Pan Q., Zeng H., Guo Z., Dong W., Huang J., Lin T. (2011). MicroRNA-125b suppresses the development of bladder cancer by targeting E2F3. Int. J. Cancer.

[85] Wu D., Ding J., Wang L., Pan H., Zhou Z., Zhou J., Qu P. (2013). MicroRNA-125b inhibits cell migration and invasion by targeting matrix metallopeptidase 13 in bladder cancer. Oncol. Lett..

[86] Zhao X., He W., Li J., Huang S., Wan X., Luo H., Wu D. (2015). MiRNA-125b inhibits proliferation and migration by targeting SphK1 in bladder cancer. Am. J. Trans. Res..

[87] Jia A.Y., Castillo-Martin M., Bonal D.M., Sanchez-Carbayo M., Silva J.M., Cordon-Cardo C. (2014). MicroRNA-126 inhibits invasion in bladder cancer via regulation of ADAM9. Br. J. Cancer.

[88] Xiao J., Lin H.Y., Zhu Y.Y., Zhu Y.P., Chen L.W. (2016). MiR-126 regulates proliferation and invasion in the bladder cancer BLS cell line by targeting the PIK3R2-mediated PI3K/Akt signaling pathway. OncoTargets Ther..

[89] Zhou X.U., Qi L., Tong S., Cui Y.U., Chen J., Huang T., Chen Z., Zu X.B. (2015). MiR-128 downregulation promotes growth and metastasis of bladder cancer cells and involves VEGF-C upregulation. Oncol. Lett..

[90] Majid S., Dar A.A., Saini S., Shahryari V., Arora S., Zaman M.S., Chang I., Yamamura S., Chiyomaru T., Fukuhara S. (2012). MicroRNA-1280 inhibits invasion and metastasis by targeting ROCK1 in bladder cancer. PLoS ONE.

[91] Lv M., Zhong Z., Chi H., Huang M., Jiang R., Chen J. (2016). Genome-wide screen of miRNAs and targeting mRNAs reveals the negatively regulatory effect of miR-130b-3p on PTEN by PI3K and integrin β1 signaling pathways in bladder carcinoma. Int. J. Mol. Sci..

[92] Zhou Y., Wu D., Tao J., Qu P., Zhou Z., Hou J. (2013). MicroRNA-133 inhibits cell proliferation, migration and invasion by targeting epidermal growth factor receptor and its downstream effector proteins in bladder cancer. Scand. J. Urol..

[93] Sun D.K., Wang J.M., Zhang P., Wang Y.Q. (2015). MicroRNA-138 regulates metastatic potential of bladder cancer through ZEB2. Cell. Physiol. Biochem..

[94] Yonemori M., Seki N., Yoshino H., Matsushita R., Miyamoto K., Nakagawa M., Enokida H. (2016). Dual tumor-suppressors miR-139–5p and miR-139–3p targeting matrix metalloprotease 11 in bladder cancer. Cancer Sci..

[95] Wang H., Li Q., Niu X., Wang G., Zheng S., Fu G., Wang Z. (2017). MiR-143 inhibits bladder cancer cell proliferation and enhances their sensitivity to gemcitabine by repressing IGF-1R signaling. Oncol. Lett..

[96] Villadsen S.B., Bramsen J.B., Ostenfeld M.S., Wiklund E.D., Fristrup N., Gao S., Hansen T.B., Jensen T.I., Borre M., Orntoft T.F. (2012). The miR-143/-145 cluster regulates plasminogen activator inhibitor-1 in bladder cancer. Br. J. Cancer.

[97] Guo Y., Ying L., Tian Y., Yang P., Zhu Y., Wang Z., Qiu F., Lin J. (2013). MiR-144 downregulation increases bladder cancer cell proliferation by targeting EZH2 and regulating Wnt signaling. FEBS J..

[98] Chiyomaru T., Enokida H., Tatarano S., Kawahara K., Uchida Y., Nishiyama K., Fujimura L., Kikkawa N., Seki N., Nakagawa M. (2010). MiR-145 and miR-133a function as tumour suppressors and directly regulate FSCN1 expression in bladder cancer. Br. J. Cancer.

[99] Noguchi S., Yamada N., Kumazaki M., Yasui Y., Iwasaki J., Naito S., Akao Y. (2013). socs7, a target gene of microRNA-145, regulates interferon-β induction through STAT3 nuclear translocation in bladder cancer cells. Cell Death Dis..

[100] Kou B., Gao Y., Du C., Shi Q., Xu S., Wang C.Q., Wang X., He D., Guo P. (2014). MiR-145 inhibits invasion of bladder cancer cells by targeting PAK1. Urol. Oncol..

[101] Zhu Z., Xu T., Wang L., Wang X., Zhong S., Xu C., Shen Z. (2014). MicroRNA-145 directly targets the insulin-like growth factor receptor I in human bladder cancer cells. FEBS Lett..

[102] Matsushita R., Yoshino H., Enokida H., Goto Y., Miyamoto K., Yonemori M., Inoguchi S., Nakagawa M., Seki N. (2016). Regulation of UHRF1 by dual-strand tumor-suppressor microRNA-145 (miR-145-5p and miR-145-3p): Inhibition of bladder cancer cell aggressiveness. Oncotarget.

[103] Xiang W., Wu X., Huang C., Wang M., Zhao X., Luo G., Li Y., Jiang G., Xiao X., Zeng F. (2017). PTTG1 regulated by miR-146–3p promotes bladder cancer migration, invasion, metastasis and growth. Oncotarget.

[104] Huang B., Zhai W., Hu G., Huang C., Xie T., Zhang J., Xu Y. (2016). MicroRNA-206 acts as a tumor suppressor in bladder cancer via targeting YRDC. Am. J. Trans. Res..

[105] Hirata H., Hinoda Y., Ueno K., Shahryari V., Tabatabai Z.L., Dahiya R. (2012). MicroRNA-1826 targets VEGFC, β-catenin (CTNNB1) and MEK1 (MAP2K1) in human bladder cancer. Carcinogenesis.

[106] Yao K., He L., Gan Y., Zeng Q., Dai Y., Tan J. (2015). MiR-186 suppresses the growth and metastasis of bladder cancer by targeting NSBP1. Diagn. Pathol..

[107] Lv L., Li Y., Deng H., Zhang C., Pu Y., Qian L., Xiao J., Zhao W., Liu Q., Zhang D. (2015). MiR-193a-3p promotes the multi-chemoresistance of bladder cancer by targeting the HOXC9 gene. Cancer Lett..

[108] Zhang M., Zhuang Q., Cui L. (2016). MiR-194 inhibits cell proliferation and invasion via repression of RAP2B in bladder cancer. Biomed. Pharmacother..

[109] Lin Y., Wu J., Chen H., Mao Y., Liu Y., Mao Q., Yang K., Zheng X., Xie L. (2012). Cyclin-dependent kinase 4 is a novel target in micoRNA-195-mediated cell cycle arrest in bladder cancer cells. FEBS Lett..

[110] Zhao C., Qi L., Chen M., Liu L., Yan W., Tong S., Zu X. (2015). MicroRNA-195 inhibits cell proliferation in bladder cancer via inhibition of cell division control protein 42 homolog/signal transducer and activator of transcription-3 signaling. Exp. Ther. Med..

[111] Sakaguchi T., Yoshino H., Yonemori M., Miyamoto K., Sugita S., Matsushita R., Itesako T., Tatarano S., Nakagawa M., Enokida H. (2017). Regulation of ITGA3 by the dual-stranded microRNA-199 family as a potential prognostic marker in bladder cancer. Br. J. Cancer.

[112] Song T., Zhang X., Yang G., Song Y., Cai W. (2015). Decrement of miR-199a-5p contributes to the tumorigenesis of bladder urothelial carcinoma by regulating MLK3/NF-κB pathway. Am. J. Trans. Res..

[113] Liu L., Qiu M., Tan G., Liang Z., Qin Y., Chen L., Chen H., Liu J. (2014). MiR-200c inhibits invasion, migration and proliferation of bladder cancer cells through down-regulation of BMI-1 and E2F3. J. Trans. Med..

[114] Bo J., Yang G., Huo K., Jiang H., Zhang L., Liu D., Huang Y. (2011). MicroRNA-203 suppresses bladder cancer development by repressing bcl-w expression. FEBS J..

[115] Saini S., Arora S., Majid S., Shahryari V., Chen Y., Deng G., Yamamura S., Ueno K., Dahiya R. (2011). Curcumin modulates microRNA-203-mediated regulation of the Src-Akt axis in bladder cancer. Cancer Prev. Res..

[116] Sun X., Du P., Yuan W., Du Z., Yu M., Yu X., Hu T. (2015). Long non-coding RNA HOTAIR regulates cyclin J via inhibition of microRNA-205 expression in bladder cancer. Cell Death Dis..

[117] Wang J., Zhang X., Wang L., Yang Y., Dong Z., Wang H., Du L., Wang C. (2015). MicroRNA-214 suppresses oncogenesis and exerts impact on prognosis by targeting PDRG1 in bladder cancer. PLoS ONE.

[118] Cheng Y., Yang X., Deng X., Zhang X., Li P., Tao J., Lu Q. (2015). MicroRNA-218 inhibits bladder cancer cell proliferation, migration, and invasion by targeting BMI-1. Tumour Biol..

[119] Li P., Yang X., Cheng Y., Zhang X., Yang C., Deng X., Li P., Tao J., Yang H., Wei J. (2017). MicroRNA-218 increases the sensitivity of bladder cancer to cisplatin by targeting Glut1. Cell. Physiol. Biochem..

[120] Wang X., Wu J., Lin Y., Zhu Y., Xu X., Xu X., Liang Z., Li S., Hu Z., Zheng X. (2014). MicroRNA-320c inhibits tumorous behaviors of bladder cancer by targeting Cyclin-dependent kinase 6. J. Exp. Clin. Cancer Res..

[121] Shang C., Zhang H., Guo Y., Hong Y., Liu Y., Xue Y. (2014). MiR-320a down-regulation mediates bladder carcinoma invasion by targeting ITGB3. Mol. Biol. Rep..

[122] Wu D., Niu X., Pan H., Zhou Y., Qu P., Zhou J. (2016). MicroRNA-335 is downregulated in bladder cancer and inhibits cell growth, migration and invasion via targeting ROCK1. Mol. Med. Rep..

[123] Xu X., Chen H., Lin Y., Hu Z., Mao Y., Wu J., Xu X., Zhu Y., Li S., Zheng X. (2013). MicroRNA-409–3p inhibits migration and invasion of bladder cancer cells via targeting c-Met. Mol. Cells.

[124] Wu C.L., Ho J.Y., Chou S.C., Yu D.S. (2016). MiR-429 reverses epithelial-mesenchymal transition by restoring E-cadherin expression in bladder cancer. Oncotarget.

[125] Liu L., Zhao X., Zhu X., Zhong Z., Xu R., Wang Z., Cao J., Hou Y. (2013). Decreased expression of miR-430 promotes the development of bladder cancer via the upregulation of CXCR7. Mol. Med. Rep..

[126] Xu X., Zhu Y., Liang Z., Li S., Xu X., Wang X., Wu J., Hu Z., Meng S., Liu B. (2016). c-Met and CREB1 are involved in miR-433-mediated inhibition of the epithelial-mesenchymal transition in bladder cancer by regulating Akt/GSK-3β/Snail signaling. Cell Death Dis..

[127] Wang J., Zhao X., Shi J., Pan Y., Chen Q., Leng P., Wang Y. (2016). miR-451 suppresses bladder cancer cell migration and invasion via directly targeting c-Myc. Oncol. Rep..

[128] Chen Z., Li Q., Wang S., Zhang J. (2015). miR4855p inhibits bladder cancer metastasis by targeting HMGA2. Int. J. Mol. Med..

[129] Li S., Xu X., Xu X., Hu Z., Wu J., Zhu Y., Chen H., Mao Y., Lin Y., Luo J. (2013). MicroRNA-490–5p inhibits proliferation of bladder cancer by targeting c-Fos. Biochem. Biophys. Res. Commun..

[130] Lan G., Yang L., Xie X., Peng L., Wang Y. (2015). MicroRNA-490–5p is a novel tumor suppressor targeting c-FOS in human bladder cancer. Arch. Med. Sci..

[131] Derrien T., Johnson R., Bussotti G., Tanzer A., Djebali S., Tilgner H., Guernec G., Martin D., Merkel A., Knowles D.G. (2012). The GENCODE v7 catalog of human long noncoding RNAs: Analysis of their gene structure, evolution, and expression. Genome Res..

[132] Guil S., Esteller M. (2012). Cis-acting noncoding RNAs: Friends and foes. Nat. Struct. Mol. Biol..

[133] Lee J.T. (2012). Epigenetic regulation by long noncoding RNAs. Science.

[134] Bussemakers M.J., van Bokhoven A., Verhaegh G.W., Smit F.P., Karthaus H.F., Schalken J.A., Debruyne F.M., Ru N., Isaacs W.B. (1999). DD3: A new prostate-specific gene, highly overexpressed in prostate cancer. Cancer Res..

[135] Fang Y., Fullwood M.J. (2016). Roles, functions, and mechanisms of long non-coding RNAs in cancer. Genom. Proteom. Bioinf..

[136] Olivieri M., Ferro M., Terreri S., Durso M., Romanelli A., Avitabile C., De Cobelli O., Messere A., Bruzzese D., Vannini I. (2016). Long non-coding RNA containing ultraconserved genomic region 8 promotes bladder cancer tumorigenesis. Oncotarget.

[137] Zhao F., Lin T., He W., Han J., Zhu D., Hu K., Li W., Zheng Z., Huang J., Xie W. (2015). Knockdown of a novel lincRNA AATBC suppresses proliferation and induces apoptosis in bladder cancer. Oncotarget.

[138] Zhu H., Li X., Song Y., Zhang P., Xiao Y., Xing Y. (2015). Long non-coding RNA ANRIL is up-regulated in bladder cancer and regulates bladder cancer cell proliferation and apoptosis through the intrinsic pathway. Biochem. Biophys. Res. Commun..

[139] He A., Liu Y., Chen Z., Li J., Chen M., Liu L., Liao X., Lv Z., Zhan Y., Zhuang C. (2016). Over-expression of long noncoding RNA BANCR inhibits malignant phenotypes of human bladder cancer. J. Exp. Clin. Cancer Res..

[140] Liu Z., Wang W., Jiang J., Bao E., Xu D., Zeng Y., Tao L., Qiu J. (2013). Downregulation of GAS5 promotes bladder cancer cell proliferation, partly by regulating CDK6. PLoS ONE.

[141] Zhang H., Guo Y., Song Y., Shang C. (2017). Long noncoding RNA GAS5 inhibits malignant proliferation and chemotherapy resistance to doxorubicin in bladder transitional cell carcinoma. Cancer Chemother. Pharmacol..

[142] Li L.J., Zhu J.L., Bao W.S., Chen D.K., Huang W.W., Weng Z.L. (2014). Long noncoding RNA GHET1 promotes the development of bladder cancer. Int. J. Clin. Exp. Pathol..

[143] Amit D., Hochberg A. (2010). Development of targeted therapy for bladder cancer mediated by a double promoter plasmid expressing diphtheria toxin under the control of H19 and IGF2-P4 regulatory sequences. J. Trans. Med..

[144] Luo M., Li Z., Wang W., Zeng Y., Liu Z., Qiu J. (2013). Long non-coding RNA H19 increases bladder cancer metastasis by associating with EZH2 and inhibiting E-cadherin expression. Cancer Lett..

[145] Luo M., Li Z., Wang W., Zeng Y., Liu Z., Qiu J. (2013). Upregulated H19 contributes to bladder cancer cell proliferation by regulating ID2 expression. FEBS J..

[146] Li S., Yu Z., Chen S.S., Li F., Lei C.Y., Chen X.X., Bao J.M., Luo Y., Lin G.Z., Pang S.Y. (2015). The YAP1 oncogene contributes to bladder cancer cell proliferation and migration by regulating the H19 long noncoding RNA. Urol. Oncol..

[147] Chen M., Zhuang C., Liu Y., Li J., Dai F., Xia M., Zhan Y., Lin J., Chen Z., He A. (2016). Tetracycline-inducible shRNA targeting antisense long non-coding RNA HIF1A-AS2 represses the malignant phenotypes of bladder cancer. Cancer Lett..

[148] Yan T.H., Lu S.W., Huang Y.Q., Que G.B., Chen J.H., Chen Y.P., Zhang H.B., Liang X.L., Jiang J.H. (2014). Upregulation of the long noncoding RNA HOTAIR predicts recurrence in stage Ta/T1 bladder cancer. Tumour Biol..

[149] Heubach J., Monsior J., Deenen R., Niegisch G., Szarvas T., Niedworok C., Schulz W.A., Hoffmann M.J. (2015). The long noncoding RNA HOTAIR has tissue and cell type-dependent effects on HOX gene expression and phenotype of urothelial cancer cells. Mol. Cancer.

[150] Shang C., Guo Y., Zhang H., Xue Y.X. (2016). Long noncoding RNA HOTAIR is a prognostic biomarker and inhibits chemosensitivity to doxorubicin in bladder transitional cell carcinoma. Cancer Chemother. Pharmacol..

[151] Berrondo C., Flax J., Kucherov V., Siebert A., Osinski T., Rosenberg A., Fucile C., Richheimer S., Beckham C.J. (2016). Expression of the long non-coding RNA HOTAIR Correlates with disease progression in bladder cancer and is contained in bladder cancer patient urinary exosomes. PLoS ONE.

[152] Martinez-Fernandez M., Feber A., Duenas M., Segovia C., Rubio C., Fernandez M., Villacampa F., Duarte J., Lopez-Calderon F.F., Gomez-Rodriguez M.J. (2015). Analysis of the Polycomb-related lncRNAs HOTAIR and ANRIL in bladder cancer. Clin. Epigenetics.

[153] Wang Y.Y., Wu Z.Y., Wang G.C., Liu K., Niu X.B., Gu S., Meng J.S. (2016). LINC00312 inhibits the migration and invasion of bladder cancer cells by targeting miR-197–3p. Tumour Biol..

[154] He W., Cai Q., Sun F., Zhong G., Wang P., Liu H., Luo J., Yu H., Huang J., Lin T. (2013). Linc-UBC1 physically associates with polycomb repressive complex 2 (PRC2) and acts as a negative prognostic factor for lymph node metastasis and survival in bladder cancer. Biochim. Biophys..

[155] Zhu Y., Dai B., Zhang H., Shi G., Shen Y., Ye D. (2016). Long non-coding RNA LOC572558 inhibits bladder cancer cell proliferation and tumor growth by regulating the AKT-MDM2-p53 signaling axis. Cancer Lett..

[156] Ying L., Chen Q., Wang Y., Zhou Z., Huang Y., Qiu F. (2012). Upregulated MALAT-1 contributes to bladder cancer cell migration by inducing epithelial-to-mesenchymal transition. Mol. Biosys..

[157] Han Y., Liu Y., Nie L., Gui Y., Cai Z. (2013). Inducing cell proliferation inhibition, apoptosis, and motility reduction by silencing long noncoding ribonucleic acid metastasis-associated lung adenocarcinoma transcript 1 in urothelial carcinoma of the bladder. Urology.

[158] Han Y., Liu Y., Zhang H., Wang T., Diao R., Jiang Z., Gui Y., Cai Z. (2013). Hsa-miR-125b suppresses bladder cancer development by down-regulating oncogene SIRT7 and oncogenic long non-coding RNA MALAT1. FEBS Lett..

[159] Fan Y., Shen B., Tan M., Mu X., Qin Y., Zhang F., Liu Y. (2014). TGF-β-induced upregulation of malat1 promotes bladder cancer metastasis by associating with suz12. Clin. Cancer Res..

[160] Xue Y., Ma G., Zhang Z., Hua Q., Chu H., Tong N., Yuan L., Qin C., Yin C., Zhang Z. (2015). A novel antisense long noncoding RNA regulates the expression of MDC1 in bladder cancer. Oncotarget.

[161] Ying L., Huang Y., Chen H., Wang Y., Xia L., Chen Y., Liu Y., Qiu F. (2013). Downregulated MEG3 activates autophagy and increases cell proliferation in bladder cancer. Mol. BioSyst..

[162] He A., Chen Z., Mei H., Liu Y. (2016). Decreased expression of LncRNA MIR31HG in human bladder cancer. Cancer Biomark..

[163] Chen T., Xie W., Xie L., Sun Y., Zhang Y., Shen Z., Sha N., Xu H., Wu Z., Hu H. (2015). Expression of long noncoding RNA lncRNA-n336928 is correlated with tumor stage and grade and overall survival in bladder cancer. Biochem. Biophys. Res. Commun..

[164] Zhu Y., Yu M., Li Z., Kong C., Bi J., Li J., Gao Z., Li Z. (2011). ncRAN, A newly identified long noncoding RNA, enhances human bladder tumor growth, invasion, and survival. Urology.

[165] XianGuo C., ZongYao H., Jun Z., Song F., GuangYue L., LiGang Z., KaiPing Z., YangYang Z., ChaoZhao L. (2016). Promoting progression and clinicopathological significance of NEAT1 over-expression in bladder cancer. Oncotarget.

[166] Liu L., Liu Y., Zhuang C., Xu W., Fu X., Lv Z., Wu H., Mou L., Zhao G., Cai Z. (2015). Inducing cell growth arrest and apoptosis by silencing long non-coding RNA PCAT-1 in human bladder cancer. Tumour Biol..

[167] Zhuang C., Li J., Liu Y., Chen M., Yuan J., Fu X., Zhan Y., Liu L., Lin J., Zhou Q. (2015). Tetracycline-inducible shRNA targeting long non-coding RNA PVT1 inhibits cell growth and induces apoptosis in bladder cancer cells. Oncotarget.

[168] Zhang J., Shi Z., Nan Y., Li M. (2016). Inhibiting malignant phenotypes of the bladder cancer cells by silencing long noncoding RNA SChLAP1. Int. Urol. Nephrol..

[169] Zhao X.L., Zhao Z.H., Xu W.C., Hou J.Q., Du X.Y. (2015). Increased expression of SPRY4-IT1 predicts poor prognosis and promotes tumor growth and metastasis in bladder cancer. Int. J. Clin. Exp. Pathol..

[170] Liu D., Li Y., Luo G., Xiao X., Tao D., Wu X., Wang M., Huang C., Wang L., Zeng F. (2017). LncRNA SPRY4-IT1 sponges miR-101–3p to promote proliferation and metastasis of bladder cancer cells through up-regulating EZH2. Cancer Lett..

[171] Han Y., Liu Y., Gui Y., Cai Z. (2013). Long intergenic non-coding RNA TUG1 is overexpressed in urothelial carcinoma of the bladder. J. Surg. Oncol..

[172] Liu Q., Liu H., Cheng H., Li Y., Li X., Zhu C. (2017). Downregulation of long noncoding RNA TUG1 inhibits proliferation and induces apoptosis through the TUG1/miR-142/ZEB2 axis in bladder cancer cells. OncoTargets Ther..

[173] Tan J., Qiu K., Li M., Liang Y. (2015). Double-negative feedback loop between long non-coding RNA TUG1 and miR-145 promotes epithelial to mesenchymal transition and radioresistance in human bladder cancer cells. FEBS Lett..

[174] Iliev R., Kleinova R., Juracek J., Dolezel J., Ozanova Z., Fedorko M., Pacik D., Svoboda M., Stanik M., Slaby O. (2016). Overexpression of long non-coding RNA TUG1 predicts poor prognosis and promotes cancer cell proliferation and migration in high-grade muscle-invasive bladder cancer. Tumour Biol..

[175] Fan Y., Shen B., Tan M., Mu X., Qin Y., Zhang F., Liu Y. (2014). Long non-coding RNA UCA1 increases chemoresistance of bladder cancer cells by regulating Wnt signaling. FEBS J..

[176] Wang X., Gong Y., Jin B., Wu C., Yang J., Wang L., Zhang Z., Mao Z. (2014). Long non-coding RNA urothelial carcinoma associated 1 induces cell replication by inhibiting BRG1 in 5637 cells. Oncol. Rep..

[177] Srivastava A.K., Singh P.K., Rath S.K., Dalela D., Goel M.M., Bhatt M.L. (2014). Appraisal of diagnostic ability of UCA1 as a biomarker of carcinoma of the urinary bladder. Tumour Biol..

[178] Eissa S., Matboli M., Essawy N.O., Kotb Y.M. (2015). Integrative functional genetic-epigenetic approach for selecting genes as urine biomarkers for bladder cancer diagnosis. Tumour Biol..

[179] Eissa S., Matboli M., Essawy N.O., Shehta M., Kotb Y.M. (2015). Rapid detection of urinary long non-coding RNA urothelial carcinoma associated one using a PCR-free nanoparticle-based assay. Biomarkers.

[180] Li H.J., Li X., Pang H., Pan J.J., Xie X.J., Chen W. (2015). Long non-coding RNA UCA1 promotes glutamine metabolism by targeting miR-16 in human bladder cancer. Jpn. J. Clin. Oncol..

[181] Wang Y., Chen W., Yang C., Wu W., Wu S., Qin X., Li X. (2012). Long non-coding RNA UCA1a(CUDR) promotes proliferation and tumorigenesis of bladder cancer. Int. J. Oncol..

[182] Zhang S., Zhong G., He W., Yu H., Huang J., Lin T. (2016). lncRNA up-regulated in nonmuscle invasive bladder cancer facilitates tumor growth and acts as a negative prognostic factor of recurrence. J. Urol..

[183] Zhuang J., Lu Q., Shen B., Huang X., Shen L., Zheng X., Huang R., Yan J., Guo H. (2015). TGFβ1 secreted by cancer-associated fibroblasts induces epithelial-mesenchymal transition of bladder cancer cells through lncRNA-ZEB2NAT. Sci. Rep..

[184] Xue M., Pang H., Li X., Li H., Pan J., Chen W. (2016). Long non-coding RNA urothelial cancer-associated 1 promotes bladder cancer cell migration and invasion by way of the hsa-miR-145-ZEB1/2-FSCN1 pathway. Cancer Sci..

[185] Li Z., Li X., Wu S., Xue M., Chen W. (2014). Long non-coding RNA UCA1 promotes glycolysis by upregulating hexokinase 2 through the mTOR-STAT3/microRNA143 pathway. Cancer Sci..

[186] Peter S., Borkowska E., Drayton R.M., Rakhit C.P., Noon A., Chen W., Catto J.W. (2014). Identification of differentially expressed long noncoding RNAs in bladder cancer. Clin. Cancer Res..

[187] Cao Q., Wang N., Qi J., Gu Z., Shen H. (2016). Long noncoding RNAGAS5 acts as a tumor suppressor in bladder transitional cell carcinoma via regulation of chemokine (CC motif) ligand 1 expression. Mol. Med. Rep..

[188] Yang C., Li X., Wang Y., Zhao L., Chen W. (2012). Long non-coding RNA UCA1 regulated cell cycle distribution via CREB through PI3-K dependent pathway in bladder carcinoma cells. Gene.

[189] Chen M., Calin G.A., Meng Q.H. (2014). Circulating microRNAs as Promising Tumor Biomarkers. Adv. Clin. Chem..

[190] Fabris L., Ceder Y., Chinnaiyan A.M., Jenster G.W., Sorensen K.D., Tomlins S., Visakorpi T., Calin G.A. (2016). The potential of MicroRNAs as prostate cancer biomarkers. Eur. Urol..

[191] Weber J.A., Baxter D.H., Zhang S., Huang D.Y., Huang K.H., Lee M.J., Galas D.J., Wang K. (2010). The microRNA spectrum in 12 body fluids. Clin. Chem..

[192] Vickers K.C., Palmisano B.T., Shoucri B.M., Shamburek R.D., Remaley A.T. (2011). MicroRNAs are transported in plasma and delivered to recipient cells by high-density lipoproteins. Nat. Cell Biol..

[193] Iftikhar H., Carney G.E. (2016). Evidence and potential in vivo functions for biofluid miRNAs: From expression profiling to functional testing: Potential roles of extracellular miRNAs as indicators of physiological change and as agents of intercellular information exchange. BioEssays.

[194] Ostenfeld M.S., Jeppesen D.K., Laurberg J.R., Boysen A.T., Bramsen J.B., Primdal-Bengtson B., Hendrix A., Lamy P., Dagnaes-Hansen F., Rasmussen M.H. (2014). Cellular disposal of miR23b by RAB27-dependent exosome release is linked to acquisition of metastatic properties. Cancer Res..

[195] Jiang X., Du L., Duan W., Wang R., Yan K., Wang L., Li J., Zheng G., Zhang X., Yang Y. (2016). Serum microRNA expression signatures as novel noninvasive biomarkers for prediction and prognosis of muscle-invasive bladder cancer. Oncotarget.

[196] Jiang X., Du L., Wang L., Li J., Liu Y., Zheng G., Qu A., Zhang X., Pan H., Yang Y. (2015). Serum microRNA expression signatures identified from genome-wide microRNA profiling serve as novel noninvasive biomarkers for diagnosis and recurrence of bladder cancer. Int. J. Cancer.

[197] Fang Z., Dai W., Wang X., Chen W., Shen C., Ye G., Li L. (2016). Circulating miR-205: A promising biomarker for the detection and prognosis evaluation of bladder cancer. Tumour Biol..

[198] Du M., Shi D., Yuan L., Li P., Chu H., Qin C., Yin C., Zhang Z., Wang M. (2015). Circulating miR-497 and miR-663b in plasma are potential novel biomarkers for bladder cancer. Sci. Rep..

[199] Pospisilova S., Pazourkova E., Horinek A., Brisuda A., Svobodova I., Soukup V., Hrbacek J., Capoun O., Hanus T., Mares J. (2016). MicroRNAs in urine supernatant as potential non-invasive markers for bladder cancer detection. Neoplasma.

[200] Fendler A., Stephan C., Yousef G.M., Kristiansen G., Jung K. (2016). The translational potential of microRNAs as biofluid markers of urological tumours. Nat. Rev. Urol..

[201] Pan S., Ryu S.Y., Sheu S.S. (2011). Distinctive characteristics and functions of multiple mitochondrial Ca^2+^ influx mechanisms. Sci. China Life Sci..

[202] Weber T.A., Reichert A.S. (2010). Impaired quality control of mitochondria: Aging from a new perspective. Exp. Gerontol..

[203] Poyton R.O., McEwen J.E. (1996). Crosstalk between nuclear and mitochondrial genomes. Annu. Rev. Biochem..

[204] Mercer T.R., Neph S., Dinger M.E., Crawford J., Smith M.A., Shearwood A.M., Haugen E., Bracken C.P., Rackham O., Stamatoyannopoulos J.A. (2011). The human mitochondrial transcriptome. Cell.

[205] Sripada L., Tomar D., Singh R. (2012). Mitochondria: One of the destinations of miRNAs. Mitochondrion.

[206] Kren B.T., Wong P.Y., Sarver A., Zhang X., Zeng Y., Steer C.J. (2009). MicroRNAs identified in highly purified liver-derived mitochondria may play a role in apoptosis. RNA Biol..

[207] Latronico M.V., Condorelli G. (2012). The might of microRNA in mitochondria. Circ. Res..

[208] Barrey E., Saint-Auret G., Bonnamy B., Damas D., Boyer O., Gidrol X. (2011). Pre-microRNA and mature microRNA in human mitochondria. PLoS ONE.

[209] Warburg O. (1956). On respiratory impairment in cancer cells. Science.

[210] Zheng J. (2012). Energy metabolism of cancer: Glycolysis versus oxidative phosphorylation. Oncol. Lett..

[211] Fogg V.C., Lanning N.J., Mackeigan J.P. (2011). Mitochondria in cancer: At the crossroads of life and death. Chin. J. Cancer.

[212] Jiang S., Zhang L.F., Zhang H.W., Hu S., Lu M.H., Liang S., Li B., Li Y., Li D., Wang E.D. (2012). A novel miR-155/miR-143 cascade controls glycolysis by regulating hexokinase 2 in breast cancer cells. EMBO J..

[213] Fang R., Xiao T., Fang Z., Sun Y., Li F., Gao Y., Feng Y., Li L., Wang Y., Liu X. (2012). MicroRNA-143 (miR-143) regulates cancer glycolysis via targeting hexokinase 2 gene. J. Biol. Chem..

[214] Tomasetti M., Nocchi L., Staffolani S., Manzella N., Amati M., Goodwin J., Kluckova K., Nguyen M., Strafella E., Bajzikova M. (2014). MicroRNA-126 suppresses mesothelioma malignancy by targeting IRS1 and interfering with the mitochondrial function. Antioxid. Redox Signaling.

[215] Li G., Li X.P., Jiang L., Lu J., Liu X., Chen S.J. (2009). Silencing of COX-2 in nasopharyngeal carcinoma cells with a shRNAmir lentivirus vector. J. South. Med. Univ..

[216] Ritterson Lew C., Guin S., Theodorescu D. (2015). Targeting glycogen metabolism in bladder cancer. Nat. Rev. Urol..

[217] Fei X., Qi M., Wu B., Song Y., Wang Y., Li T. (2012). MicroRNA-195–5p suppresses glucose uptake and proliferation of human bladder cancer T24 cells by regulating GLUT3 expression. FEBS Lett..

[218] Takai T., Yoshikawa Y., Inamoto T., Minami K., Taniguchi K., Sugito N., Kuranaga Y., Shinohara H., Kumazaki M., Tsujino T. (2017). A Novel Combination RNAi toward warburg effect by replacement with miR-145 and Silencing of PTBP1 Induces apoptotic cell death in bladder cancer cells. Int. J. Mol. Sci..

[219] Bochenek G., Hasler R., El Mokhtari N.E., Konig I.R., Loos B.G., Jepsen S., Rosenstiel P., Schreiber S., Schaefer A.S. (2013). The large non-coding RNA ANRIL, which is associated with atherosclerosis, periodontitis and several forms of cancer, regulates ADIPOR1, VAMP3 and C11ORF10. Hum. Mol. Genet..

[220] Yang F., Zhang H., Mei Y., Wu M. (2014). Reciprocal regulation of HIF-1α and lincRNA-p21 modulates the Warburg effect. Mol. Cell.

